# The circadian clock modulates anti-cancer properties of curcumin

**DOI:** 10.1186/s12885-016-2789-9

**Published:** 2016-09-29

**Authors:** Ashapurna Sarma, Vishal P. Sharma, Arindam B. Sarkar, M. Chandra Sekar, Karunakar Samuel, Michael E. Geusz

**Affiliations:** 1 Department of Biological Sciences, Bowling Green State University, 217 Life Science Building, Bowling Green, OH 43403 USA; 2 Life Sciences Institute, University of Michigan, Ann Arbor, MI 48109 USA; 3 Department of Pharmaceutical Sciences, University of Findlay, Findlay, OH 45840 USA

**Keywords:** Circadian rhythm, Glioma, Curcumin, Curcuminoids, Chronopharmacology, Apoptosis, Cell cycle

## Abstract

**Background:**

Curcuminoids of the spice turmeric and their enhanced derivatives have much potential as cancer treatments. They act on a wide variety of biological pathways, including those regulating cell division and circadian rhythms. It is known that circadian clocks can modify cancer therapy effectiveness, according to studies aimed at optimizing treatments based on the circadian cycle. It is therefore important to determine whether treatments with curcumin or similar chemotherapeutic agents are regulated by circadian timing. Similarly, it is important to characterize any effects of curcumin on timing abilities of the circadian clocks within cancer cells.

**Methods:**

We examined the circadian clock’s impact on the timing of cell death and cell division in curcumin-treated C6 rat glioma cells through continuous video microscopy for several days. To evaluate its persistence and distribution in cancer cells, curcumin was localized within cell compartments by imaging its autofluorescence. Finally, HPLC and spectroscopy were used to determine the relative stabilities of the curcumin congeners demethoxycurcumin and bisdemethoxycurcumin that are present in turmeric.

**Results:**

Circadian rhythms in cell death were observed in response to low (5 μM) curcumin, reaching a peak several hours before the peak in rhythmic expression of mPER2 protein, a major circadian clock component. These results revealed a sensitive phase of the circadian cycle that could be effectively targeted in patient therapies based on curcumin or its analogs. Curcumin fluorescence was observed in cell compartments at least 24 h after treatment, and the two congeners displayed greater stability than curcumin in cell culture medium.

**Conclusions:**

We propose a mechanism whereby curcuminoids act in a sustained manner, over several days, despite their tendency to degrade rapidly in blood and other aqueous media. During cancer therapy, curcumin or its analogs should be delivered to tumor cells at the optimal phase for highest efficacy after identifying the circadian phase of the cancer cells. We confirmed the greater stability of the curcumin congeners, suggesting that they may produce sustained toxicity in cancer cells and should be considered for use in patient care.

**Electronic supplementary material:**

The online version of this article (doi:10.1186/s12885-016-2789-9) contains supplementary material, which is available to authorized users.

## Background

Curcumin is a promising phytochemical for treating several cancers. This ingredient of the spice turmeric has been used to treat several ailments for thousands of years because of its anti-inflammatory, anti-microbial, and wound-healing properties [1]. Curcumin’s anti-cancer properties have much potential when used alone or in combination with standard chemotherapies or radiation treatments. It arrests tumor cell proliferation by inhibiting multiple signal transduction pathways, interfering with the cell cycle, and inducing apoptosis. Relative to most agents currently used to target cancer cells, curcumin is reported to have low toxicity towards normal cells [2, 3]. The molecular targets of curcumin and related curcuminoids include several transcription factors, oncogenes, and signaling proteins engaged in cancer initiation and progression [4, 5]. For example, NF-kB (nuclear factor-kB) and AP-1 (activator protein-1) are constitutively active in cancer cells, and curcumin enhances apoptosis by blocking signaling pathways that rely on these transcription factors [2, 6]. Curcumin has multiple molecular targets through which it causes cell toxicity in various types of cancers, including gliomas [5, 7–16]. Additionally, curcumin used in combination with cisplatin or doxorubicin, common chemotherapy drugs, induces apoptosis in glioblastoma cell lines [15].

One weakness of curcumin as a cancer treatment is its poor bioavailability and rapid degradation in the body. Nevertheless, the two major congeners in the turmeric root, demethoxycurcumin (DMC) and bisdemethoxycurcumin (BDMC) may persist longer in tissues than curcumin. DMC, BDMC, and the major curcumin metabolite tetrahydrocurcumin could provide a sustained anti-cancer effect [17–19].

Another temporal factor influencing the efficacy of cancer therapies is the circadian rhythm that is prominent in many physiological processes [20, 21]. The circadian timing system generates approximately 24-h rhythms in the body through autonomous intracellular circadian clocks, including ones identified in many cancer cells [22, 23]. The role of these clocks in cancer is not yet known, although circadian-regulated proteins appear to alter tumor growth rate [24–27]. As the molecular mechanism of circadian clocks is explored in greater depth, chemotherapy regimens based on circadian rhythms are being developed and tested [28].

The molecular circadian timing within clock cells could be altered by curcumin treatments. One likely curcumin target, *Bmal1*, is a critical gene within the molecular oscillator producing circadian rhythms. *Bmal1* is activated by curcumin through stimulation of PPAR-γ [29, 30]. Studies also suggest that polyphenols such as curcumin activate sirtuin 1 (SIRT1), which also regulates circadian rhythms. SIRT1, a histone deacetylase, indirectly controls the circadian clock by (1) down-regulating NF-kB [31]; (2) inhibiting nuclear localization of the clock protein mPER2 through deacetylation of the tumor suppressor PML [32]; and (3) binding to the CLOCK-BMAL1 dimer, promoting deacetylation and degradation of mPER2 [33]. Thus, curcumin could alter circadian rhythms in normal and cancer cells, although there are no reported effects on the circadian timing mechanism.

Drug chronotherapy (use of circadian timing to optimize pharmacokinetics or pharmacodynamics) is an effective medical approach [34]. Many proteins involved in drug absorption, metabolism or elimination display daily oscillations in synthesis or activity. Studies with rodents show differing effects and toxicities from chemotherapeutic drugs depending on time of day of administration [35]. Reported circadian regulation of chemotherapeutic treatments includes anticancer drugs 5-flurouracil, doxorubicin, roscovitine, and platinum complex analogs cisplatin, carboplatin, and oxaliplatin [36–38]. Some but not all of these effects likely depend on the ability of circadian clocks to regulate daily cell division timing. The most effective time of day when chemotherapies based on curcumin should be administered to patients is unknown. In this study, we identified a phase of the circadian cycle when a low dose of curcuminoids is most effective at inducing death of rat glioma cancer cells in vitro, and we found that circadian rhythms in gene expression persist at this dosage.

## Methods

### Cell culture

Rat C6 glioma cells were cultured in Dulbecco's Modified Eagle Medium (DMEM) containing penicillin (100 units/ml), streptomycin (100 μg/ml), 10 % fetal bovine serum (FBS), and no pyruvate or phenol red (complete medium). Cells were grown in 100-mm tissue culture dishes at 37 °C in 5 % CO_2_ and were passaged when they were nearly confluent. Cells were cotransfected with a construct producing a fusion protein of mPER2 and firefly luciferase (*mPer2::mPer2:luc*) along with *CMV::neo* [39]. These bioluminescent reporter gene cells were used in most experiments.

### Bioluminescence assay

C6 cells containing the *mPer2::mPer2:luc* reporter gene were seeded (10^5^ cells/dish) in 35-mm tissue culture dishes and incubated in DMEM medium containing 10 % FBS at 37 °C in 5 % CO_2_. When the plates were 90-100 % confluent, the cells were washed twice with a 10 mM HEPES-buffered, low-bicarbonate (4.2 mM), phenol red-free DMEM, designed for use in room air, combined with 10 % FBS, which was designated as final medium (FM), After an exchange with FM, cells were treated with 20 μM forskolin in ethanol (0.01 % v/v) for 2 h to synchronize the cellular circadian clocks. Immediately before imaging, 0.2 mM of the luciferase substrate luciferin (Xenogen) was added. For experiments with low-dose curcumin, 0.2 mM luciferin and 5 μM curcumin (CUR, Sigma-Aldrich C-1386) were added to the plate 12 h after forskolin treatment. To monitor rhythmic expression of the clock protein, bioluminescence was recorded using a Wallac Victor 1420 Multilabel plate reader (Perkin Elmer). Culture dishes were maintained at 37 °C while readings were taken hourly for 50 to 96 h. The background noise was subtracted from each reading and signals were summed across replicate cultures.

### Curcumin treatment

To examine circadian rhythms in cell division and cell death after treatment with different doses of CUR, C6 cells were seeded at a density of 6 x 10^5^ cells in 60-mm dishes containing complete medium and then grown overnight to 50 % confluency. They were then given one of three treatments: 20 μM forskolin for 2 h, forskolin for 2 h followed by 5 μM CUR, or forskolin followed by 10 μM CUR, all in FM. For forskolin treatments, cells were washed twice with FM, medium was exchanged with the forskolin treatment, and then cells were incubated for 2 h at 37 °C. CUR was dissolved in dimethyl sulfoxide (DMSO) to make a 25 mM stock solution and was diluted in FM before the treatment.

### Long-term time-lapse cell imaging

To image individual mitotic and apoptotic events a single field of view of cells maintained in FM and sealed in a 60-mm dish was captured at 5-min intervals using time-lapse videography continuously over 4–6 days. The imaging system consisted of an inverted microscope with a 20x objective lens, red LED light source, and a digital color camera. The imaging system was placed inside a 37 °C incubator to maintain ideal culture conditions. Time-lapse recording software FLIX (Nimisis.com) captured live cell events, and each image from the time series was analyzed by eye and with ImageJ software (National Institutes of Health).

### Immunocytochemistry

Immunofluorescence staining was used to identify apoptotic cells and verify the peak and trough of the circadian rhythm in apoptotic events. C6 cells were fixed in 100 % methanol for 5 min and standard immunocytochemistry methods were used to identify cleaved caspase-3-positive cells. Anti-cleaved caspase-3 primary antibody (Cell Signaling) was used at 1:1000 dilution. The samples were rinsed after overnight incubation at 4 °C, and were then incubated for 2 h with Alexa488-conjugated, complementary secondary antibody. For nuclear staining, cells were stained with Hoechst 33342 (10–20 ng/ml, Invitrogen) for 2 min. Cells were imaged with a DMI3000B inverted fluorescence microscope (Leica Microsystems), a Rolera Thunder cooled-CCD camera with a back-thinned, back-illuminated, electron-multiplying sensor (Photometrics), X-Light spinning-disk confocal unit (CrestOptics), and a Spectra X LED light engine (Lumencore) with image acquisition and processing controlled by Metamorph software (Molecular Devices). Images were collected with a 40x objective lens along with standard DAPI and fluorescein filters. Images were further analyzed with ImageJ software.

### Curcumin localization in C6 cells

For autofluorescence imaging of curcumin, C6 cells were grown in 35-mm glass-bottom dishes (MatTek) for one day, treated with 5 μM CUR in DMSO, and incubated at 37 °C. After 1 h or 24 h, cells were washed with FM and autofluorescence was imaged using standard fluorescein filters. Images were captured with the confocal fluorescence imaging system and a 63x oil immersion lens. After 24 h, additional treated cells were fixed with methanol, stained with Hoechst, and imaged using DAPI filters.

### Spectral analysis of curcumin

The absorption spectrum of 5 μM CUR in final medium with DMSO was measured with a GENESYS 10S UV–vis Spectrophotometer. In a second set of measurements, cells in 60-mm dishes were given 5 μM CUR, medium was removed 0, 4, 8, 12, 24, 48, 72 and 96 h later, and spectra were measured (scanning mode 200–600 nm). Curcumin absorbs maximally near 430 nm.

### HPLC analysis

The High Pressure Liquid Chromatography (HPLC) studies were performed with a Hewlett-Packard (HP) 1050 HPLC system and Chemstation software. The stationary phase consisted of Zorbax Eclipse Plus column (Phenyl Hexyl, 4.6 x 150 mm, 95 Å pore size, pH range 2–9, Agilent Technologies, Santa Clara, CA) along with a compatible Zorbax Eclipse Plus Phenyl Hexyl pre-column. The mobile phase was 45 % acetonitrile and 55 % buffer at pH 3.0 (1 % acetic acid). The pH was adjusted with triethanolamine (approximately 0.4 ml/L). The run time was 15 min, the column was maintained at 30 °C, flow rate was 1.0 ml/min, injection volume was 10 μl, and detection wavelength was 420 nm. Under these chromatographic conditions curcumin eluted with a retention time of 6–9 min. The elution order was BDMC < DMC < curcumin. For best results the column was conditioned by running the mobile phase with the described composition at a flow rate of 1 ml/min for one hour. The organic modifier and the buffer component used were HPLC grade.

Standards containing all three curcuminoids (curcumin, DMC, and BDMC) were prepared across a concentration range between 0.025 and 10 μg/ml in DMSO. DMSO was used in the standard curve because of curcumin’s limited stability in aqueous buffers at neutral pH. The standard curve was found to be linear across the entire range. The limit of detection for all three curcuminoids was 0.025 μg/ml, although the limit of quantitation was determined to be 0.1 μg/ml as the accuracy below 15 % of the theoretical concentration was deemed unacceptable. The precision of the method remained below 10 % of the mean at all levels for curcumin, down to 0.05 μg/ml for DMC, and down to 0.1 μg/ml for BDMC. The standard curve data are in Additional file 1.

### Data analysis

For measurements of circadian rhythms in mPER2 expression, total bioluminescence measurements from each dish were collected every hour for up to 4 days. After background subtraction, each time series was detrended by subtracting a 24-h running average. For continuous imaging of mitotic and apoptotic rates the cell counts taken from each image frame at 5-min intervals were summed into 1-h bins. The average hourly rates for each day were compared by two-tailed Student’s *t*-test and ANOVA. The dominant period within each averaged time series was found by Fast Fourier Transform (FFT) analysis (Origin, OriginLab). Circular statistics of circadian rhythms in apoptotic and mitotic events (Rayleigh test for randomness) were performed using Oriana software (Kovach Computing Services), and the mean phase vector was used to indicate the phase of rhythms. Period estimates of rhythms were determined using the Lomb-Scargle Periodogram MATLAB program LOMB, FFT, and the Maximum Entropy Method (MEM, kSpectra Toolkit, SpectraWorks). When multiple peaks were found by MEM the peak within the circadian range (19–29 h) was used as the most precise measure of the circadian rhythm.

## Results

### Circadian rhythms persist at low curcumin concentrations

The C6 rat glioma cell line was selected to test for an effect of the circadian clock on curcumin’s anti-cancer properties because it displays circadian rhythms in expression of the core circadian clock gene *mPer2* in cell cultures [40] and in tumorsphere cultures [39]. Curcumin decreases NF-kB activation, inhibits C6 cell proliferation, and induces cell death. We treated C6 cells with a low dose of curcumin (CUR) to cause limited but significant cell death over several days while allowing enough cells to remain for measurements [40]. Also, because curcumin acts on many intracellular signaling pathways [41] it was important to use a dosage that would not suppress the molecular mechanism of the circadian oscillator.

We used C6 cells stably transfected with a reporter gene that generates a fusion protein of mPER2 and firefly luciferase under control by the *mPer2* gene promoter [39]. Circadian clock cells in cultures of these *mPer2::mPer2:luc* C6 cells were synchronized with a 2-h forskolin treatment. Starting 12 h later, to allow acute forskolin effects to subside, medium was exchanged with medium containing curcumin dissolved in 0.02 or 0.04 % DMSO for 5 and 10 μM curcumin, respectively. The DMSO concentration was well below the threshold of 10 % for toxic effects on colon cancer cells [42], and the threshold of 0.1 %, below which solutions can be injected safely into the vitreous of the rat eye without affecting retinal neurons [43]. This pulse of curcumin was intended to mimic a single delivery of the drug to a cancer patient either intravenously or intracerebrally. Similar to what occurs in the body, it was expected to be degraded in vitro over the next few hours based on the known properties of curcumin [44, 45].

**Fig. 1:**
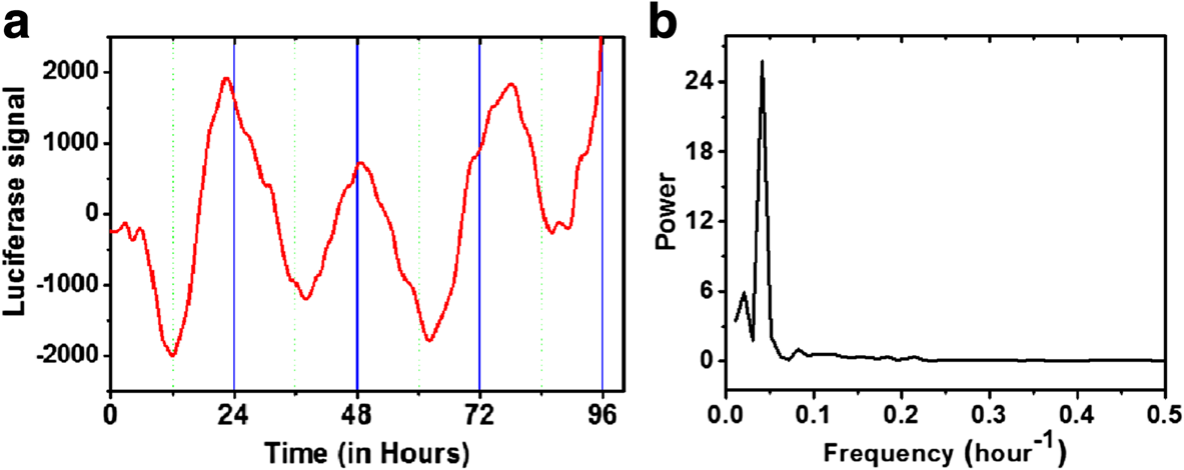
Circadian rhythms in *mPer2* clock gene expression persist after treatment with 5 μM curcumin. (**a**) Signal from the *mPer2::mPer2:luc* reporter gene after a forskolin pulse used to synchronize cellular circadian oscillators is shown as the hourly integrated light signal (relative light units averaged from 4 independent cultures). (**b**) A significant period of 24.48 h was detected by Lomb-Scargle Periodogram analysis (*p* < 0.001) is shown in the frequency spectrum

C6 cell cultures in medium with 10 % FBS are reported to have a 23.5-h circadian rhythm when measured with a destabilized luciferase reporter gene controlled by the *mPer2* promoter [40]. Similarly, in a previous study we detected a 25.2-h bioluminescence rhythm in a C6 culture expressing the mPER2-LUC fusion protein, according to Lomb-Scargle Periodogram (LS) analysis (*p* < 0.001) [46]. The *mPer2::mPer2:luc* C6 cells also expressed circadian rhythms in cultures given 5 μM CUR (Fig. 1a). For cultures treated with 5 μM CUR the average period was estimated as 24.48 h by LS (*p* < 0.001) (Fig. 1b) and 24.47 h by FFT.

### Long-term effects of curcumin on mitosis and cell death

Because circadian rhythms persisted after low-dose curcumin treatments, we synchronized the circadian clock cells in C6 cultures and examined the pattern of individual mitotic and apoptotic events for any effects from curcumin or the circadian clock. To determine whether the 5 μM curcumin treatment was sufficient to produce anticancer effects on C6, and whether a higher dose would be more useful for this study, C6 cells were monitored continuously by digital video imaging of cell cultures. To identify ongoing cell division and cell death events in cultures time-lapse imaging (TLI) was performed using 5-min intervals between frames for up to 5 days, after synchronizing cells with forskolin and then treating with curcumin 12 h later. All events were counted from a single field-of-view which represented the cell events occurring in the dish (Additional file 2). The culture dish and microscope remained for days in a sealed incubator without disturbance. During TLI, there was initially an average of 23.57 ± 7.03 (SD) cells in the field-of-view (range 18 to 30, *n* = 15 cultures).

**Fig. 2:**
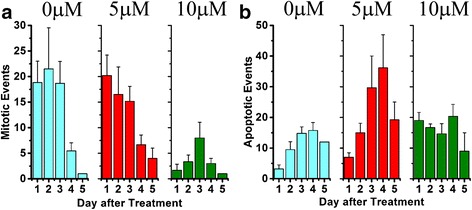
Effects of curcumin on mitosis and apoptosis of C6 glioma cells. Suppression of mitotic rate (**a**) and induction of cell death rate (**b**) at two curcumin concentrations. Average hourly rates were imaged in single fields-of-view for 5 days after a forskolin pulse and a single curcumin treatment

Distinct mitotic and apoptotic events were visible and counted following exposure to 0, 5, and 10 μM CUR (Fig. 2). When mitotic events during day 1 of imaging were compared, 10 μM was significantly more effective at suppressing cell division than 5 μM (ANOVA, *F* = 4.537, Fisher post hoc test, *p* = 0.0148) and the control (*p* = 0.0216) (Fig. 2a). The 5 μM group was, however, not significantly different from the control (*p* = 0.806). When the total mitotic events for the first four days were examined, 10 μM again resulted in a significant suppression of mitosis (*p* = 0.0485) relative to control (0 μM: 62.67 ± 36.439, *n* = 6; 5 μM: 60.00 ± 30.470, *n* = 6; 10 μM: 15.00 ± 0.358, *n* = 3). By day 4 (5^th^ day in culture), cell confluence in the control dishes limited our ability to detect cell division events, so they were not counted.

When the apoptotic events occurring in the 0, 5, and 10 μM groups during day 1 were compared, 10 μM produced significant cell death (*F* = 18.751, *p* < 0.001), but not 5 μM (Fig. 2b). However, when total apoptotic events over days 1–4 were compared (F = 6.398, *p* = 0.0128) the 5 μM treatment caused significant cell death (*p* = 0.00384). Cell death rates were overall lower in 10 μM-treated cells, and more cell death occurred in the first day.

### Circadian modulation of curcumin effects on mitosis and cell survival

**Fig. 3:**
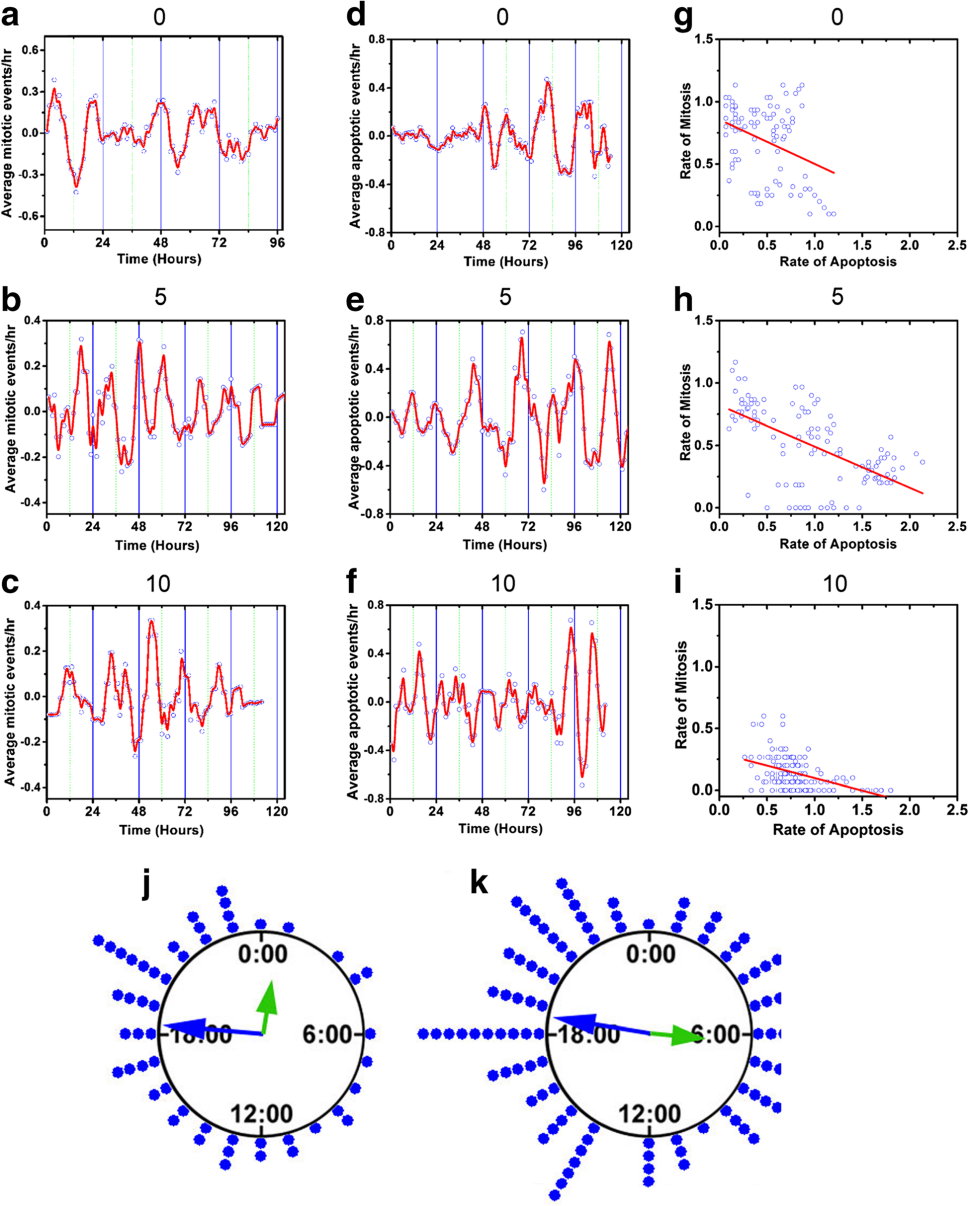
Circadian clock regulation of curcumin efficacy. **a**-**c**: Mitotic events in C6 cultures showed circadian rhythms in 0 and 10 μM but not 5 μM CUR. **d**-**f**: Cell death rates (apoptotic events/hr) displayed circadian rhythms in 0 and 5 but not 10 μM CUR (blue circles: events, red line: after adjacent averaging). **g**-**i**: Apoptotic and mitotic rates were inversely correlated as shown by linear regressions. **j**, **k**: The phase of cell death events displayed significant clustering (*p* < 0.001) in 5 μM CUR on days 2 and 3 (left and right, Z = 14.62 and 7.399 by Rayleigh Test, respectively). Long arrow indicates phase of mean vector. Short arrow (green) indicates peak of mPER2::LUC rhythm from Fig. 1. The curcumin treatment began at 12:00 AM (0:00) on Day 1. Each dot indicates 2 events

To determine whether ongoing events of cell division and cell death in cultures exposed to curcumin are modulated by circadian timing, we measured the period and phase of any significant circadian rhythms (Fig. 3). Because individual events within the field-of-view were few the data were pooled from all cultures in each treatment group. According to LS analysis, a significant circadian rhythm in mitosis was detected in the control culture (Table 1). Most rhythms were similarly identified by FFT.

**Table 1:** Period analysis of C6 cell cultures

Curcumin Treatment (μM)	Imaging Duration (hrs)	Mitosis			Apoptosis		
		LS	FFT	MEM	LS	FFT	MEM
0	114	22.6	21.3	21.3	18.8	21.3	18.9
5	135	14.9	15.0	Non-circadian	22.3	23.3	24.4
10	112	18.5	18.3	20.5	11.1	14.2	Non-circadian

All period estimates are in hours. LS: Lomb-Scargle Periodogram, FFT: Fast Fourier Transform, MEM: Maximum Entropy Method. All LS results were significant (*p* < 0.001). “Non-circadian” indicates neither of the two most powerful peaks of the frequency spectra fell in the circadian range (19–29).

To provide a more precise estimate of the circadian period than what LS or FFT can provide we used the Maximum Entropy Method to find periods with greater resolution. According to MEM the untreated cultures displayed an average period of 21.3 h for mitotic events. This rhythm in cell division was similar to the doubling time of 22 h reported for C6 cells [47], suggesting that the forskolin treatment may have synchronized individual cell cycles. The 10 μM CUR-treated cultures displayed a 20.5-h period. Thus, circadian rhythms were observed in the mitotic events of the control and 10 μM cultures (Fig. 3a, c), but not in the 5 μM group (Fig. 3b). In the presence of 5 μM CUR the cell division cycles and circadian rhythms appeared to be uncoupled. Mitosis displayed a rhythm of about 15 h, and these shorter ultradian rhythms (defined here as having periods less than 18 h) may have resulted from curcumin acting on cell cycle oscillations. To further test the periods of these cultures, we analyzed the mitotic events with FFT, which yielded periods of 21.3 h for the control group, 15.0 h for 5 μM, and 18.3 h for the 10 μM group.

Apoptotic events occurring in the cell cultures were analyzed to detect any circadian rhythms. A rhythm with a period of 18.9 h was detected in the untreated group (Fig. 3d), which is at the edge of a typical circadian range of 19–30 h (Table 1). The apoptotic events occurring in 5 μM CUR-treated cells followed a circadian rhythm with a period of 22.3 h (Fig. 3e). All the period estimates for this treatment group fell in the circadian range (Table 1). Circadian rhythms were absent in 10 μM CUR-treated cells (Fig. 3f), which instead had an ultradian rhythm of 11–14 h (Table 1). Circadian rhythms in apoptosis persisted in the 5 but not 10 μM cultures, indicating that the clock can modulate cell death at the lower curcumin dosage.

Because mitotic and apoptotic rates appeared to reach their maxima at different times, we examined this relationship in the untreated, 5, and 10 μM groups. The apparent inverse relationship between apoptotic and mitotic events was confirmed by a linear correlation test comparing the two time-series data sets: Pearson’s correlation *r* values were −0.350, −0.599, and −0.437 for the 0, 5, and 10 μM treatments, respectively (Fig. 3g-i). The mitotic rate was highest in the untreated group as apoptotic events were fewer in that group, while the 10 μM group had lower mitotic rates and higher rates of apoptosis during the initial days of treatment.

Along with the period analyses, we also examined the phase relationships between the mPER2 rhythm shown in Fig. 1 and the mitotic and apoptotic rhythms. We applied circular statistics to identify significant clustering of apoptotic events in curcumin-treated groups over the first three 24-h cycles of imaging. When examining the timing of these events relative to the forskolin treatment, significant clustering was observed at 18.3 and 18.6 h during the 2^nd^ and 3^rd^ days of imaging, respectively, in the 5 μM group (Fig. 3j, k). When comparing these phases with the mPER2 rhythm, they occurred on the rising phase, about 6 and 11 h before the corresponding circadian peaks in mPER2 protein expression.

There was no significant clustering of cell death events in the 10 μM group, in agreement with the loss of circadian periodicity of apoptosis. The phase of cell deaths in the control group (0 μM) was significantly clustered (*p* < 0.05) on the second day, but the mean vector was not significantly different from that of the 5 μM group, indicating that curcumin did not produce a measurable phase shift of the rhythm (control: 16:24 with a 99 % confidence interval of 12:31 and 20:17 h on days 2 and 3, respectively; 5 μM: 18:18 with 99 % confidence intervals of 16:34 and 20:03 h on days 2 and 3). There was no significant clustering of apoptotic or mitotic events in the remaining groups.

**Fig. 4:**
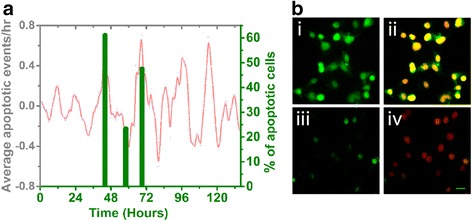
Expression of activated caspase-3 according to phase of the circadian cycle. **a**: Percentage of apoptotic cell counts at 45, 57, and 69 h after adding 5 μM CUR. The relative changes in the percent cell death agreed with the peaks and trough in the circadian rhythm of death rate for cells treated with 5 μM CUR (red line, from Fig. 3e, shown here for comparison). **b**: Immunostaining cleaved caspase-3 in apoptotic cells (green) at two circadian phases, the peak at 45 h (i) and the trough at 57 h (iii), after 5 μM CUR treatment; ii and iv: The same cells merged with Hoechst nuclear staining (red). Scale bar = 10 μm

As an additional test of whether cell death events vary according to the circadian cycle, we quantified the percentage of cells expressing activated caspase-3, a late apoptotic marker [48, 49] in C6 cells given 5 μM CUR. Three times were selected to coincide with the second peak, the following trough, and the third peak observed in the rhythm in apoptosis (Fig. 3e). The three phases examined showed relative differences in cell death matching the oscillations in apoptotic events in the time series (Fig. 4a). The percentage of apoptotic cells was 61.15 ± 0.03 % at the 45^th^ hr and 47.62 ± 0.04 % at the 69^th^ hr, which are both peak phases in the circadian rhythm of death rate. During the trough phase (57^th^ hr) the percentage of apoptotic cells declined to 23.50 ± 0.02 %. Visibly, there were more cells stained with anti-caspase3 antibody at the peak phases (Fig. 4b, I and II) than at a trough phase (Fig. 4b, III and IV).

### Stability and localization of curcuminoids and their metabolites

**Fig. 5:**
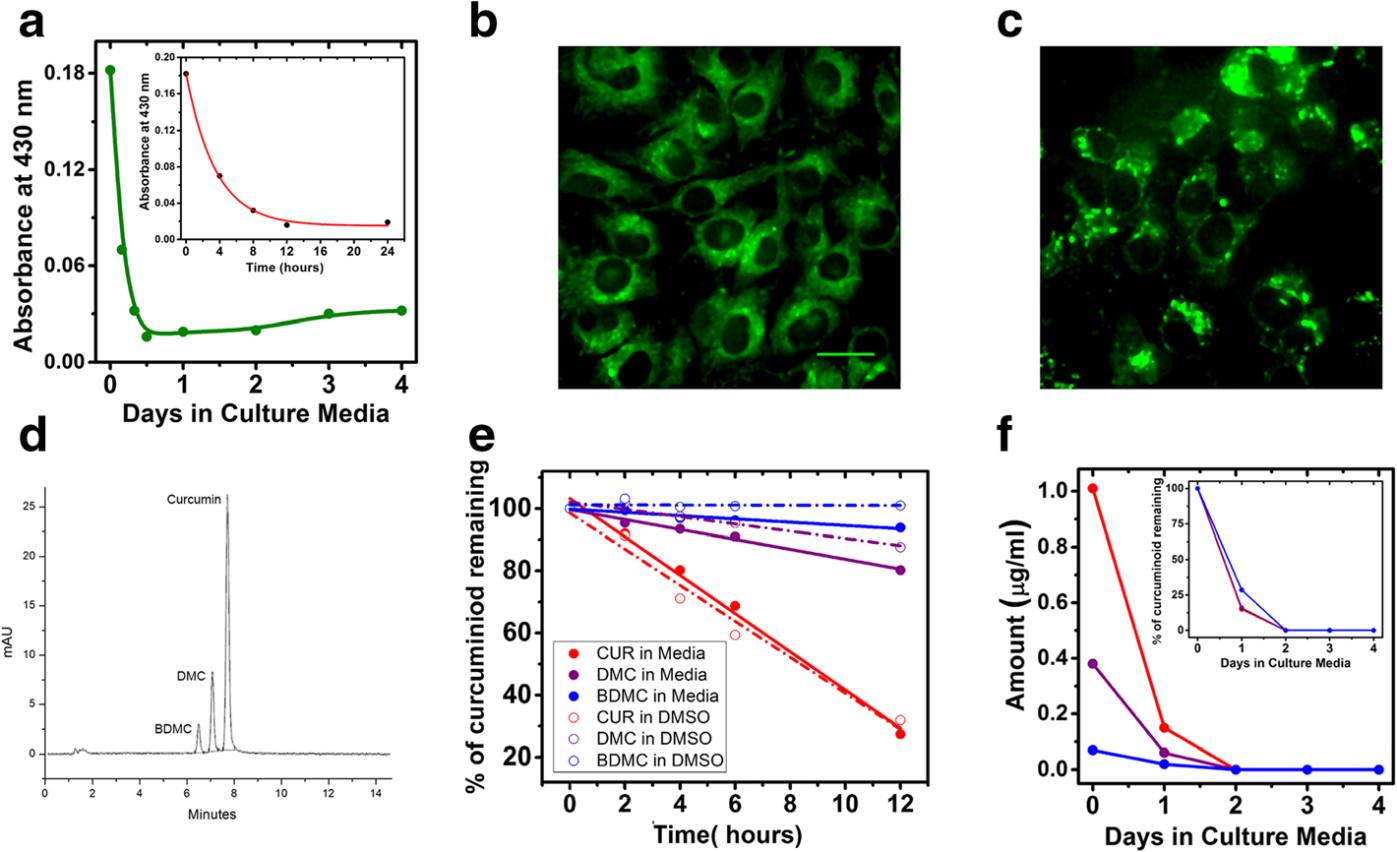
Stability of the 3 curcuminoids in cell culture. Curcumin degraded with a half-life of about 1.7 h in cell culture medium with cells present. **a**: Curcumin absorbance in culture medium with C6 cells for 4 days measured with a spectrophotometer. Inset: Absorbance during the first 24 h. **b**: 5 μM CUR produced fluorescence in live C6 cells within 1 h after its introduction. Scale bar = 5 μm. **c**: Curcuminoid fluorescence persisted in cells for at least 24 h (same scale as B). **d**: A representative HPLC chromatogram showing complete baseline separation of the 3 curcuminoids from cell culture medium containing 0.3 % DMSO. The first group of merged peaks near 1.6 min represents the chromophoric compounds from the media. The second peak at 6.45 min is BDMC, the peak at 7.04 min is that of DMC, and the last peak at 7.68 min is that of curcumin. (mAU = milliabsorbance units). This is the same CUR preparation used for treating the cells. **e**: HPLC measurements show curcuminoids DMC and BDMC persist longer than curcumin in cell culture medium and degrade even slower in DMSO. **f**: HPLC measurements of curcuminoids in culture medium with C6 cells present for 5 days. Inset: The same data normalized to initial levels. Line colors are as in **e.**

Although curcumin is being tested as an anticancer drug in a number of clinical trials [5, 50], its use is limited because of fast degradation at neutral and alkaline pH and poor tissue absorption [51, 52]. Studies have shown that curcumin is relatively more stable in culture media containing 10 % fetal bovine serum (FBS), compared to phosphate buffer or culture media without FBS [53]. Despite the expected loss of curcumin, our TLI data showed that apoptosis continued for several days after initial treatment with 5 or 10 μM CUR. Using a spectrophotometer we found evidence of curcumin in culture media during the days the cells were imaged (Fig. 5). A standard curve was created at curcumin’s maximal absorbance near 430 nm [54]. Nevertheless, curcumin levels declined within the first day, becoming nearly undetectable (Fig. 5a). During the first 24 h, the curcumin declined with a half-life of about 1.7 h by degrading or entering cells (Fig. 5a inset). The curcumin levels in media also showed an unexpected small increase after the second day.

Autofluorescence imaging of curcumin’s cellular distribution in live cells showed that curcumin is present in the nucleus one hour after application (Fig. 5b). Although curcumin levels decreased considerably in the culture media, curcuminoids were visible in C6 cells for at least 24 h after application (Fig. 5c). The autofluorescence study was performed in live C6 cells 1 h and 24 h after curcumin treatment. To confirm the nuclear localization of curcumin 24 h after curcumin treatment, cells were fixed and stained with Hoechst nuclear stain (Additional file 3). It was observed that curcumin was present in the nucleus and was concentrated in distinct intra-nuclear sites, as described previously [55].

The curcumin treatment used in the study contained the additional congeners DMC and BDMC (Fig. 5d), which may have contributed to the apoptotic or mitotic effects. The relative amount of curcumin present in the CUR treatment was similar to that reported by the manufacturer (≥65 %). To better understand the potential contributions of the congeners to the cell effects we used HPLC to measure curcumin, DMC, and BDMC concentrations in cell culture medium at 0, 2, 4, 6 and 12 h after treatment. Samples containing curcuminoids in complete medium (10 % FBS serum and 0.3 % DMSO) were preserved at −20 °C in the dark. To prevent further degradation each sample was allowed to thaw before 10 μl was injected without further purification or delay.

As predicted from previous studies [56], the HPLC results indicated that curcumin degraded by over 75 % within 12 h in culture medium at room temperature, but the two congeners degraded more slowly (Fig. 5e), around 20 % for DMC and only 8 % for BDMC, suggesting that they could have been responsible for cell death along with curcumin after the first day of treatment. The degradation rates of the congeners in DMSO were relatively slower than in culture media (Fig. 5e). Degradation patterns of the three curcuminoids were also measured in samples of medium from cultures containing C6 cells once per day for 4 days after the curcumin treatment (Fig. 5f). All conditions of these samples were kept similar to those of the previous TLI cultures. There was a rapid decline of curcumin in culture medium during the first 24 h at 37 °C (to about 15 %). DMC decreased to about 16 % in the first 24 h of treatment, whereas BDMC declined to about 28 % (Fig. 5f inset).

Although the culture medium had very little curcumin or congener present, the cells retained curcuminoids, as shown in Figs. 5b and c, which could have been responsible for apoptosis and other cellular effects. To determine whether the medium retains an anti-cancer property after curcumin levels decline, we examined C6 cells treated with a conditioned medium (CM) that was withdrawn from a C6 culture one day after treatment with 5 μM curcumin (Additional file 4). Significant cell death or mitotic arrest was not observed in response to CM treatment.

## Discussion

This is the first study to examine the effects of curcumin on circadian rhythms in cancer cells and whether the circadian clock is altered by curcumin treatments. Curcumin impacts several signaling pathways regulating the intracellular timing cycles generating circadian rhythms. These targets include STAT, PPARγ, and NFkB that act on gene expression within the two interconnected molecular timing loops of the circadian oscillator that generate the rhythm. Nevertheless, the circadian rhythm in C6 cells persisted following the 5 μM curcumin treatment that produced increased cell death days later. Although this dosage did not arrest the clock, the 10 μm curcumin treatment did produce a loss of any detectable circadian rhythm in apoptosis. The absence of circadian periodicity at 10 μM could have been caused by disruption of individual circadian oscillators within clock cells or the coupling between circadian clocks. Nevertheless, fast circadian oscillations in mitotic events persisted.

Perhaps the best explanation for these results is that the clock regulates cell death by increasing the probability of its occurrence at a particular phase of the circadian cycle, but treatment dosages that produce greater stimulation of the apoptotic pathway mask clock control of these events. Because the circadian rhythm in BMAL1 activity is considered a major timing output of the clock, producing rhythms in many genes, we speculate that it also modulates expression of proteins that degrade or transport curcumin [57]. It is unclear why the circadian rhythm in mitotic events was lost after applying 5 μM but not 10 μM curcumin. Nevertheless, the 5 μM data did show a pattern resembling a disrupted population of circadian oscillators, perhaps because of fragmentation of the previously synchronized cell population into two or more rhythmic groups. Both of these concentrations were below the reported IC_50_ of 25 μM for curcumin effects on C6 cells [58], and cells continued to proliferate during the several days of imaging, which was our intention.

Curcumin produced a delayed and persistent cell death well after it was no longer easily detectable in medium by HPLC or absorbance spectroscopy at the end of the first day after delivery. There are several possible mechanisms that can explain this sustained effect: The initial treatment altered some of the cells in a way that arrested their cell cycle and caused them to die much later. Curcumin is known to induce cell death through mitotic arrest [59], at which point cell death might begin long after the time when untreated cells would have divided. Alternatively, the cells may have continued to divide, but the treatment caused a change in the viability of the cells that was also present in the progeny of the treated cells. For example, curcumin can produce epigenetic alterations that may promote cancer cell death [60].

Another intriguing possibility is that because curcumin is lipophilic it was entrapped in cell membranes where it was protected from degradation and then released into the cytosol, perhaps through membrane turnover, thereby killing cells much later. A similar mechanism was proposed for effects from vanillin, a curcumin degradation product with weaker anti-cancer properties [61]. This possibility is supported by the curcumin autofluorescence we observed in live cells at least 24 h after exposure.

We also considered the possibility that the two congeners of curcumin may have caused the delayed cell death along with curcumin or in its absence. Both DMC and BDMC persisted much longer in culture medium than curcumin, and both are reported to have anti-cancer effects, as shown in lung cancer cell lines [62, 63]. Curcumin degrades rapidly in blood and perhaps also cerebrospinal fluid, but the more persistent congeners may be responsible for the systemic effects reported in some animal studies [64]. Interestingly, BDMC also inhibits cancer cell metastasis [65]. DMC was reported to oxidize more slowly than curcumin at physiological pH, persisting for at least a day, but BDMC was resistant to oxidation [66], suggesting that it may have a more prolonged effect on C6 cells than the other two curcuminoids. It is also possible that some or much of the curcumin autofluorescence we and others observed in cell membranes actually originated from the congeners, which have excitation and emission spectra similar to those of curcumin [67]. By passing through this cell reservoir, the congeners might have produced delayed cell toxicity. The two curcumin congeners showed greater stability in DMSO than in cell culture medium suggesting that the nonpolar cell membrane environment might also protect curcuminoids longer than culture medium or other aqueous solutions.

Finally, we did not investigate curcumin’s degradation products in C6 cells, but some of these have known anti-cancer properties [62, 68, 69]. For example, tetrahydrocurcumin is an early product that would be formed in aqueous media, such as after intravascular injection [64], and it may have contributed to cell death in the C6 cultures. Similarly, the more lipophilic degradation products may have accumulated in cell membranes, producing a delayed response. Because the medium conditioned with C6 cells for 24 h did not increase cell death, any curcumin, congeners, or degradation products present in the medium after the first day are not likely to have had much effect on cell survival. Instead, curcuminoids bound to or integrated into cell membranes are the most likely causal agents.

The C6 mitotic and apoptotic events displayed a significant inverse relationship in 0, 5, and 10 μM curcumin, indicating a coordinated timing between these two properties in cultures. Circadian clocks can control the cell division cycle at several checkpoints, most notably through p21 regulation by the core clock protein BMAL1 [70, 71]. This coupling is, however, variable as shown previously when the two types of rhythms were manipulated in Lewis lung carcinoma cells to oscillate with different periods [72]. Unlike circadian clocks, metabolic and hormonal signals can readily arrest or initiate mitosis, and the greater lability of the cell cycle period impairs its ability to provide accurate timing. Furthermore, the circadian clock is compensated to maintain a more constant period as temperature changes. We detected about a 2-h difference in period between mitotic and apoptotic oscillations in the control culture suggesting that the two rhythmic processes are not tightly coupled. Nevertheless, the correlations we detected indicate that the phase of the cell cycle can provide an estimate of circadian phase in some cancer cell types and may be useful for predicting when curcumin is most effective. In the C6 cultures, cell death was observed most often when mitotic rates were minimal.

The highest apoptotic rate was about 6–11 h before the peak of mPER2 expression in C6 cells, suggesting that there is a time of day when curcumin treatment would be most effective for treating patients. To use this result to optimize delivery of curcumin, encapsulated curcumin or curcumin nanoparticles [73] it will be necessary to predict the circadian phase of the cancer cells within the tumor. Additional studies are needed to determine whether human tumors with a functioning circadian clock are entrained by the body’s daily rhythms in cortisol, melatonin or other signals, resulting in a predictable maximum in PER2 expression for timing delivery of curcumin or similar drugs. However, any daily rhythms in curcumin’s absorption and degradation also need to be considered.

Curcumin analogs are more stable alternatives to curcumin [74] and should be tested to determine whether they too are most effective at this circadian phase. Furthermore, chemically distinct chemotherapeutic agents that act through pathways blocked by curcumin, such as NFkB [58], may be most effective at this phase in gliomas and other cancer cell types. Curcumin’s reported ability to prevent cancers may also be optimal at the time of day corresponding with this phase of highest sensitivity, which may be estimated from PER2 circadian rhythms measured in non-cancer cells of healthy individuals.

## Conclusion

Glioma cells are most vulnerable to the lethal effects of curcuminoids at a particular phase of the circadian cycle. Curcuminoids bind to cell membranes and accumulate in cell nuclei, possibly serving as a reservoir providing delayed release and sustained anti-cancer effects. Cancer patient care should include time-of-day curcumin dosing, to exploit the sensitive phase, and should consider treatment with the curcumin congeners because of their stability under physiologically relevant conditions.

## Additional files

Additional file 1: Table S1. Curcuminoid analysis (standard curve parameters). Cited in HPLC analysis section of the Methods. (PDF 95 kb)
Additional file 2: Effects of curcumin on cell division and cell death rates. Cited in long-term time-lapse cell imaging section of the Results. Shows individual frames from videos of C6 mitosis and cell death. (PPTX 1258 kb)
Additional file 3: Autofluorescence of curcumin in C6 cells. Cited in the Results section. Microscope images of curcumin autofluorescence in C6 cells 24 h after exposure. (PPTX 966 kb)
Additional file 4: Effects on C6 cells from CUR-conditioned medium. Cited in the Results section. Shows the lack of a significant effect from 24-h conditioned medium. (PPTX 161 kb)

## Abbreviations

ANOVA: Analysis of variance
AP-1: Activator protein-1
BDMC: Bisdemethoxycurcumin
CCD: Charge-coupled device
CM: Conditioned medium
CUR: Curcumin
DAPI: 4',6-diamidino-2-phenylindole dihydrochloride
DMC: Demethoxycurcumin
DMEM: Dulbecco's Modified Eagle Medium
DMSO: Dimethyl sulfoxide
FBS: Fetal bovine serum
FFT: Fast Fourier Transform
FM: Final medium
HEPES: N-2-hydroxyethylpiperazine-N'-2'-ethanesulfonic acid
HPLC: High pressure liquid chromatography
LED: Light-emitting diode
LS: Lomb-Scargle
MEM: Maximum Entropy Method
NF-kB: Nuclear factor-kB
PML: Promyelocytic leukemia protein
PPAR-γ: Peroxisome proliferator-activated receptor-γ
SIRT1: Sirtuin 1
TLI: Time-lapse imaging
