# Supplementary material for: The circadian clock modulates anti-cancer properties of curcumin
Source: BMC Cancer. 2016 Sep 29;16:759. doi: 10.1186/s12885-016-2789-9 (PMC5041585; doi:10.1186/s12885-016-2789-9)
Supplement: Additional file 1: Table S1. — Curcuminoid analysis (standard curve parameters). Cited in HPLC analysis section of the Methods. (PDF 95 kb) [file 12885_2016_2789_MOESM1_ESM.pdf]

Additional File 1: Table showing curcuminoid analysis (standard curve parameters).

Compound Concentration (μg/ml)	Standard Curve Equation	Correlation Coefficient	Accuracy (% of theoretical)	Precision (% of mean)
**CUR**				
10	y = 62.317x - 0.5767	R^2 = 0.999	100.3	2.3
5			98.7	2.3
1			99.3	3.2
0.1			108.1	2.9
0.05			129.9	9.8
0.025			153.3	8.4
**DMC**				
10	y = 66.666x - 0.4327	R^2 = 0.999	100.3	2.2
5			99	2.3
1			95.6	3.4
0.1			107.4	7
0.05			121.6	7.4
0.025			143.7	11.7
**BDMC**				
10	y = 81.224x - 1.0844	R^2 = 0.999	100.3	2.2
5			98.9	2.3
1			98.8	3.2
0.1			111.5	8.7
0.05			123.9	11.8
0.025			157	16.9
