# Supplementary material for: The circadian clock modulates anti-cancer properties of curcumin
Source: BMC Cancer. 2016 Sep 29;16:759. doi: 10.1186/s12885-016-2789-9 (PMC5041585; doi:10.1186/s12885-016-2789-9)
Supplement: Additional file 2: — Effects of curcumin on cell division and cell death rates. Cited in long-term time-lapse cell imaging section of the Results. Shows individual frames from videos of C6 mitosis and cell death. (PPTX 1258 kb) [file 12885_2016_2789_MOESM2_ESM.pptx]

## Slide 1
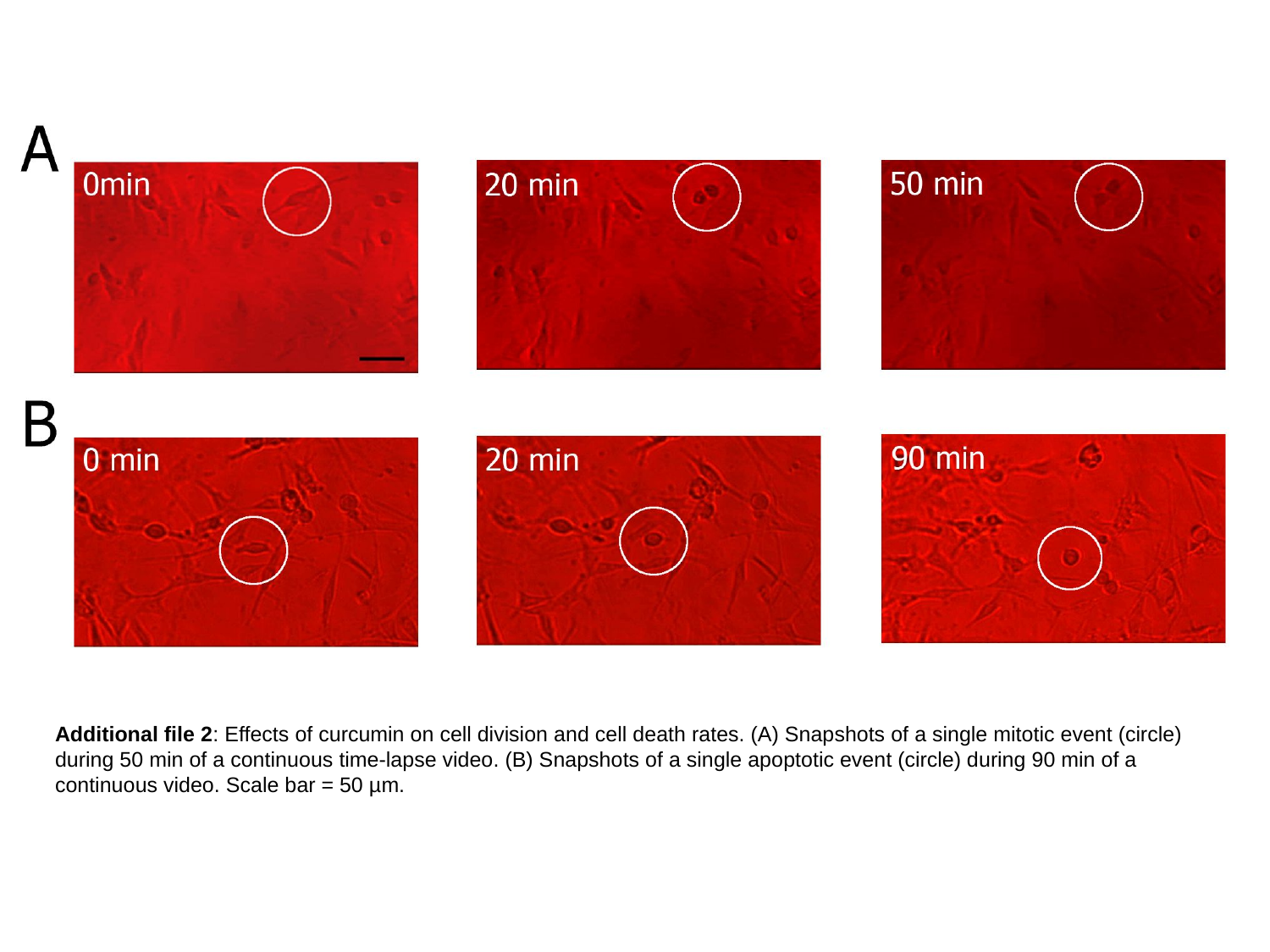

Additional file 2: Effects of curcumin on cell division and cell death rates. (A) Snapshots of a single mitotic event (circle) during 50 min of a continuous time-lapse video. (B) Snapshots of a single apoptotic event (circle) during 90 min of a continuous video. Scale bar = 50 µm.
