# Supplementary material for: The circadian clock modulates anti-cancer properties of curcumin
Source: BMC Cancer. 2016 Sep 29;16:759. doi: 10.1186/s12885-016-2789-9 (PMC5041585; doi:10.1186/s12885-016-2789-9)
Supplement: Additional file 3: — Autofluorescence of curcumin in C6 cells. Cited in the Results section. Microscope images of curcumin autofluorescence in C6 cells 24 h after exposure. (PPTX 966 kb) [file 12885_2016_2789_MOESM3_ESM.pptx]

## Slide 1
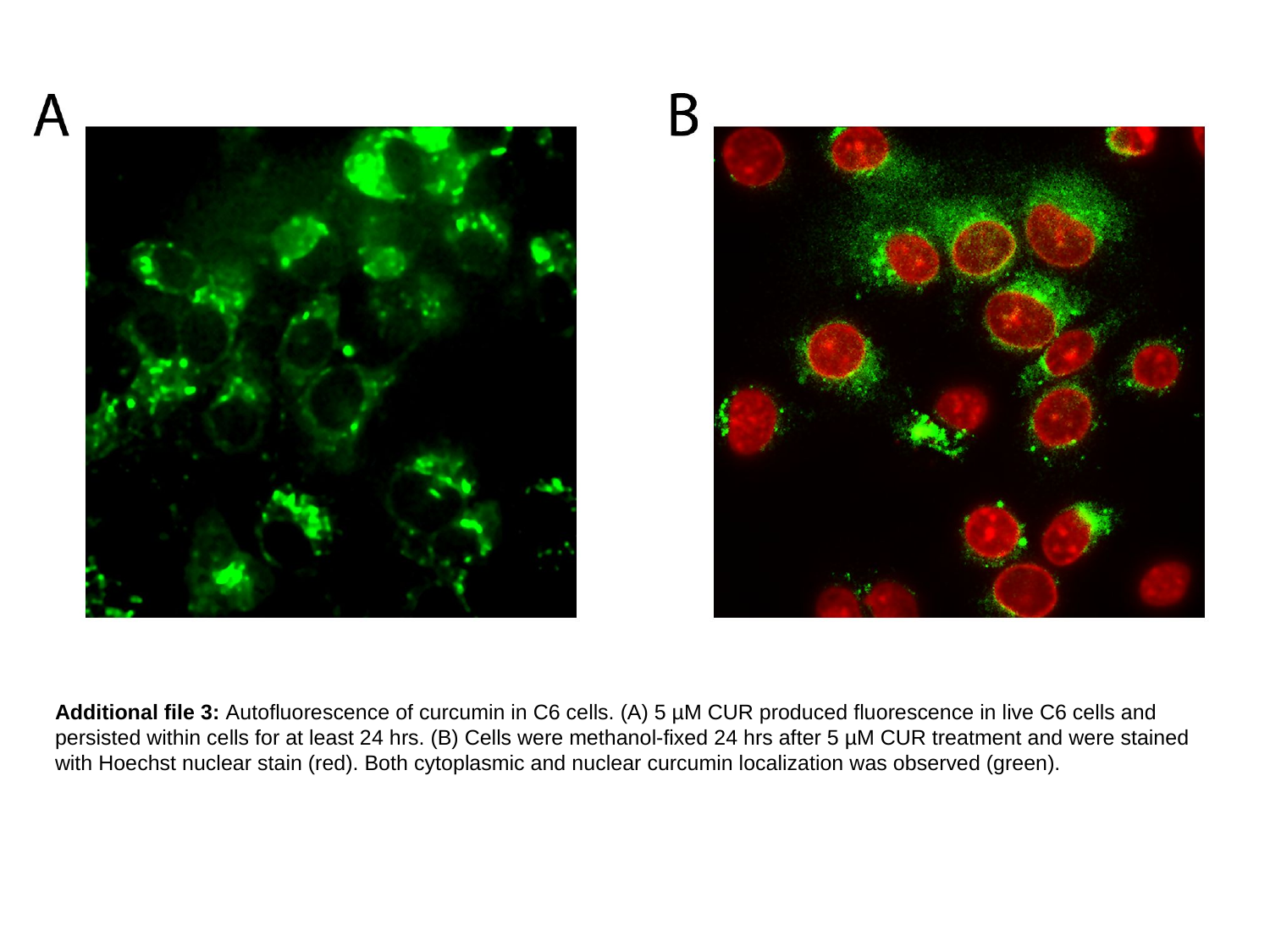

Additional file 3: Autofluorescence of curcumin in C6 cells. (A) 5 µM CUR produced fluorescence in live C6 cells and persisted within cells for at least 24 hrs. (B) Cells were methanol-fixed 24 hrs after 5 µM CUR treatment and were stained with Hoechst nuclear stain (red). Both cytoplasmic and nuclear curcumin localization was observed (green).
