# Supplementary material for: The circadian clock modulates anti-cancer properties of curcumin
Source: BMC Cancer. 2016 Sep 29;16:759. doi: 10.1186/s12885-016-2789-9 (PMC5041585; doi:10.1186/s12885-016-2789-9)
Supplement: Additional file 4: — Effects on C6 cells from CUR-conditioned medium. Cited in the Results section. Shows the lack of a significant effect from 24-h conditioned medium. (PPTX 161 kb) [file 12885_2016_2789_MOESM4_ESM.pptx]

## Slide 1
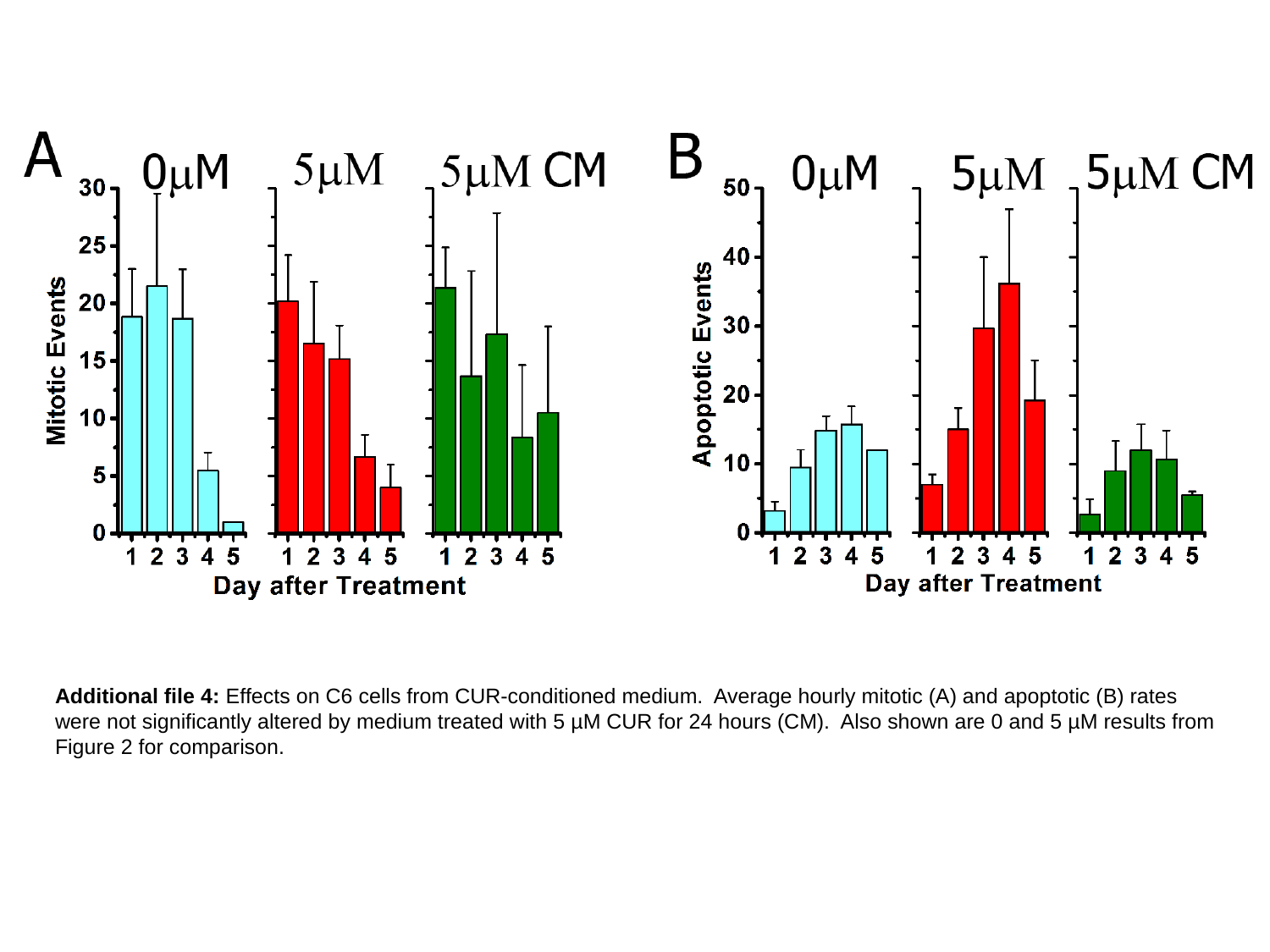

Additional file 4: Effects on C6 cells from CUR-conditioned medium. Average hourly mitotic (A) and apoptotic (B) rates were not significantly altered by medium treated with 5 µM CUR for 24 hours (CM). Also shown are 0 and 5 µM results from Figure 2 for comparison.
